# Evaluation of two strategies for debriefing simulation in the development of skills for neonatal resuscitation: a randomized clinical trial

**DOI:** 10.1186/s13104-018-3831-6

**Published:** 2018-10-17

**Authors:** Oscar Andrés Gamboa, Sergio Iván Agudelo, María Jose Maldonado, Diana C. Leguizamón, Sandra M. Cala

**Affiliations:** School of Medicine, Specialization Program in Pediatrics, Universidad de La Sabana, Campus Universitario Puente del Común, km. 7, Autopista Norte de Bogotá, Edifico H, oficina 204 D, Chía, Cundinamarca Colombia

**Keywords:** Cardiopulmonary resuscitation, Newborn, Infant, Simulation training, Team resuscitation, Debriefing

## Abstract

**Objective:**

To evaluate two *debriefing* strategies for the development of neonatal resuscitation skills in health professionals responsible for the critical newborn care in a high-complexity university Hospital.

**Results:**

A simple blind randomized clinical trial was conducted. Twenty-four professionals (pediatricians, nurses, and respiratory therapists) were randomly assigned for two interventions; one group received oral *debriefing* and the other oral *debriefing* assisted by video. Three standardized clinical scenarios that were recorded on video were executed. A checklist was applied for the evaluation, administered by a reviewer blinded to the assignment of the type of *debriefing*. The two *debriefing* strategies increased the technical and behavioral neonatal resuscitation skills of the participants, without one being superior to the other. The coefficient of the difference in the compliance percentage between the two types of *debriefing* was − 3.6% (95% CI − 13.77% to 6.47%). When comparing the development of technical and behavioral skills among the professionals evaluated, no significant differences were found between the types of *debriefing*. The two *debriefing* strategies increase compliance percentages, reaching or approaching 100%.

*Trial Registration* ClinicalTrials.gov NCT03606278. July 30, 2018. Retrospectively registered

**Electronic supplementary material:**

The online version of this article (10.1186/s13104-018-3831-6) contains supplementary material, which is available to authorized users.

## Introduction

Neonatal resuscitation is an event of high complexity and stress for the teams responsible for critical neonatal care [1]. Performing neonatal resuscitation skills correctly is associated with greater survival of the newborn [2]. Simulation-based education it a teaching method used with healthcare personnel in charge of neonatal resuscitation [1, 3]. It facilitates learning by allowing the incorporation of knowledge in a controlled environment, which provides security [4] because it standardizes the exposure to a specific event, allows the teaching of specific clinical skills, increases self-confidence, and improves the clinical judgment of the student [5, 6]. *Debriefing* is an essential component of the teaching process based on simulation [7–9]. The debrief session occurs immediately after simulation and can be guided by an instructor and structured as reflective oral and video-assisted methods [7, 10]. These two strategies are the most commonly used methods in neonatal resuscitation simulation training [11]. The literature does not provide evidence about which of the two strategies is better for use in the training of teams of health professionals responsible for the care of the newborn in a high-complexity scenarios.

The present study aims to evaluate two *debriefing* strategies, a structured instructor-guided oral *debriefing* compared with a structured instructor-guided oral *debriefing* assisted by video, in the development of neonatal resuscitation skills in health professionals responsible for the care of the newborn in a high-complexity university Hospital.

## Main text

### Methods

A simple blind randomized clinical trial was conducted. Staff for the care of critical newborn of the Clinica Universidad de La Sabana were invited to participate. Professional nurses, respiratory therapists, and pediatricians were included. The Medical Ethics Committee of the Clinic approved the protocol. The participants signed an informed consent statement.

Participants received a study material and completed a theoretical exam; the day of study and prior to randomization, were trained in individual skill stations in the steps of neonatal resuscitation. Subsequently, they immersed themselves in three standardized simulated scenarios: newborn with perinatal asphyxia, general management of the preterm newborn, and newborn with meconium. All scenarios were recorded on video and audio, to conduct structured *debriefings* in the group with video and for the evaluation of subsequent performance with a checklist, was performed by an evaluator blinded to the assignment of the *debriefing* type. Each scenario was followed immediately by an instructor-guided *debriefing* session (Additional file 1: Figure S1).

#### Randomization

The participants were randomized at two points. At the first point, they were randomly assigned to the type of *debriefing* (oral or video), stratified by the type of health professional. For this randomization, the random function of Excel was used, sorting the participants in a random number order and assigning the first half to the oral group and the other half to the video-assisted group. For the conformation of the teams, a second randomization was performed, in which the professionals within an assigned group (oral or video-assisted) were randomized to form a resuscitation team. In this randomization, Excel’s random function was also used, sorting the participants in a random number order and assigning the one that was ordered first to team 1, the second to team 2, and so on, until completing four teams per intervention, each with three professions: pediatrician, professional nurse, and respiratory therapist. The same team was maintained during the participation of each scenario (Additional file 1: Figure S1).

#### Interventions

*Structuring of the debriefing* The *debriefing* session in both groups was led by an instructor and with an assigned time of 15 min. Before starting the session, the instructor explained the learning objectives of the scenario and how the feedback process would be conducted; each session was developed in three stages. An initial stage of a descriptive type, in which each participant was encouraged to recount what they had lived and experienced, commenting in a group with their peers about the experiences they had perceived, clarifying how the events unfolded, verifying the appropriate decisions and the errors committed in the scenario and the ways they could have solved them and corrected them. Then, they proceeded to a second analytical phase, where the participant reflected on what occurred in the scenario, commenting on how their feelings were involved in the development of the case. Finally, transference phase, in which the group was encouraged to draw conclusions, realizing an application of this experience in a real-life.

*Control group* The control group was assigned to the oral *debriefing*. This group received the *debriefing* process, supported essentially by the mental search of their memories of what occurred.

*Intervention group* The intervention group was assigned to the *debriefing* assisted by video. This video was used to highlight specific points that were not easily recognized by the participants. The video was stop and rewinded as necessary.

*Evaluation*: A checklist of individual performance and by profession was constructed that included cognitive/technical and behavioral aspects (Additional files 1: Appendix S1). A reviewer blinded to the assignment of the type of debriefing by reviewing the video applied this checklist. Each item of the tool was assigned a score of 1 if the evaluated activity was correctly performed, 0 if it was not performed correctly, and N/A if it did not apply for the profession. A compliance percentage of the activities evaluated by participant in the tool was obtained, adding the points obtained onto the possible total score.

#### Sample size

A sample size was calculated using the information published in the study by Luna et al. [12]. The normal asymptotic method was used in the estimation, for expected improvements of 33% with oral *debriefing* and 90% with video-assisted *debriefing*, a type I error of 5%, a type II error of 20%, an allocation rate of 1, and two tails, obtaining a sample size of 22 participants (11 for each intervention). Finally, a sample size of 24 participants was used (12 for each intervention), which allowed organizing resuscitation teams of three participants each, for a total of four teams per *debriefing* type.

#### Statistical analysis

Descriptive analyses were performed. Box-and-whisker plots were constructed for the compliance percentage, for each profession and in each of the scenarios evaluated. To compare the *debriefing* methods, a generalized estimating equations model was constructed, which is used to develop regression models in correlated data that come from repeated measurements of the same individual over time, as was the case. The dependent variable was the compliance percentage, and the independent variables were the type of *debriefing*, the scenario, the profession, and the group. The analyses were performed using the Stata^®^ 10 program.

### Results

**Fig. 1:**
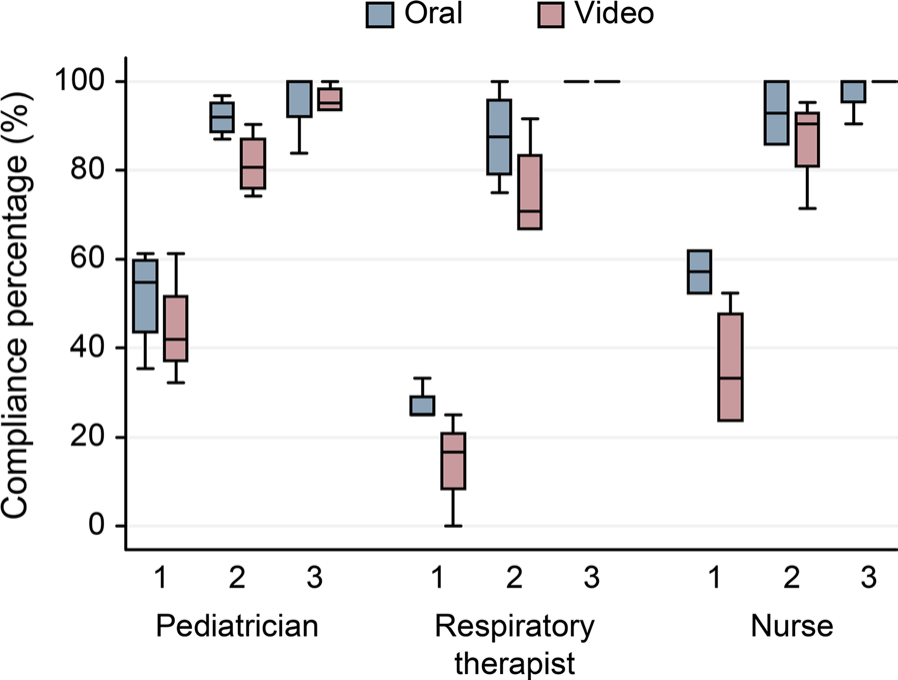
Box-and-whisker plot. Comparative illustration of performance by scenarios (1, 2, 3) and roles (pediatrician, respiratory therapist, professional nurse)

Table 1 provides the characterization data of the participant. In the box-and-whisker plot (Fig. 1), it was observed that both strategies improved skills in neonatal resuscitation, approaching 100% compliance in the third scenario evaluated. No significant differences were found between the two *debriefing* strategies. The coefficient of the difference in the compliance percentage between the two types of *debriefing* was − 3.6% (95% CI − 13.77% to 6.47%). When comparing the development of technical and behavioral skills, no significant differences were found between the types of *debriefing*, with − 6.34% (95% CI − 19.93% to 7.25%) and − 0.19% (95% CI − 10.85% to 10.46%), respectively.

**Table 1:** Characterization of the participating population

Variable	Oral *debriefing* (n = 12)	Video-assisted *debriefing* (n = 12)	p value
Average age in years (SD)	36.25 (7.78)	35.08 (7.5)	0.71
Sex n (%)			0.62
Men	2 (17)	3 (25)	
Women	10 (83)	9 (75)	
Average experience in years (SD)	7.6 (7.86)	7.6 (6.72)	1
Distribution by services n (%)			0.22
NICU	4 (33)	7 (58)	
Other^a^	8 (67)	5 (42)	

*NICU* neonatal intensive care unit

^a^Emergencies (video: 4, oral: 4), neonatal adaptation rooms (video: 1, oral: 1), surgery rooms (video: 0, oral: 2), hospitalization (video: 0, oral: 1)

The participants of the video-assisted group started at a lower point regardless of profession (Fig. 1). Given this finding, the increase in the compliance score for each type of *debriefing* was determined after each scenario, and it was found that the participants of the oral group increased their score by 26.29% (95% CI 20.44% to 32.14%) after each *debriefing* episode, while those of the video group increased by 33.55% (95% CI 27.9% to 39.2%). When performing this same analysis by profession, it was found that the two *debriefing* strategies increased their compliance percentages and that the therapists increased their scores by the greatest percentages after each scenario (Table 2).

**Table 2:** Comparison of the effect according to profession

Model	Video (95% CI)	Oral (95% CI)
All professions	33.55% (27.9% to 39.2%)	26.29% (20.44% to 32.14%)
Physicians	25.81% (18.31% to 33.3%)	22.18% (13.19% to 31.16%)
Nurses	32.14% (22.99% to 41.29%)	20.24% (13.29% to 27.19%)
Therapists	42.71% (33.92% to 51.5%)	36.46% (26.28% to 46.64%)

### Discussion

The two strategies improved the overall skills and differentiated as cognitive, technical, and behavioral in the different professions, without significant statistical differences between the two. These findings are consistent with what has been found by other authors. Sawyer et al. and Cheng et al. compared the effectiveness of video-assisted vs. oral *debriefing* on the performance of pediatric postgraduate students during a neonatal resuscitation, without showing differences between the two educational strategies [13, 14]. Fanning and Gaba reported that the advantages of using the video were not seen consistently [9].

Several factors can influence the development of competencies in *debriefing*: the type and quality of the video recording; the selection of the video segment that permits highlighting the learning objectives; the amount of video time reviewed; the time, the space, and the duration of the *debriefing*; and the expertise and knowledge of the instructor who performs the *debriefing* [7, 13]. In the systematic review of Cheng, time (long or short session) was not a factor that influenced learning [14]. In the present study, a neonatologist with expertise in neonatal resuscitation program and debriefing conducted the debriefing sessions and 5 min were given to the scenario and 15 min to the feedback session. The times of video review and mental recall were in relation to the objectives set for the scenario and the feedback session. The facilitator’s expertise in the learning objectives could have effects on the development of competencies. In relation to the use of video, the facilitator would have to better identify which segment of the video would be the best to use. However, this expertise is also important in oral feedback since the facilitator/instructor should keep in mind what data are relevant to recall so that the participants can focus on them.

The respiratory therapists had higher coefficients of improvement throughout the scenarios in both groups. It was considered that these improvements could be because they were also the group of professionals who started with lower pre-test scores. It can be stated that despite having different profiles, professions, and levels of knowledge, all achieved optimal performance thanks to their overall participation in the course.

It was not possible to compare this result with similar results since no other study has permitted the comparison of roles between different professions. In this study, the neonatal resuscitation teams were formed with the professionals exercising the role they play in the care of the newborn in the real clinical context. Other studies that have sought to evaluate the effects of simulation and *debriefing* have included a population of undergraduate medical or nursing students [12, 14], of the total number of students admitted to the study, the majority were nursing students, postgraduate medical students and medical students. In the case of studies that have sought to analyze this effect in the specific field of neonatal resuscitation, they have included pediatric and family medicine residents as study populations [15, 16]. In the present study, it was possible to evaluate the role of each profession within their own role in the neonatal resuscitation team, that is, by executing the actions for which they are prepared according to their profession and which are the ones they will develop as a member of a real-life neonatal care team.

### Conclusions

*Debriefing* shows a potential benefit for increasing the skills in neonatal resuscitation. In this study, neonatal resuscitation teams were formed that were similar to those in the real clinical context and with clinical staff having experience and expertise in advanced neonatal resuscitation. In this context, the results showed that when *debriefing* is performed immediately and assisted by a facilitator, the strategy structured by video is not superior to the oral structured strategy in the development of skills in neonatal resuscitation, including technical/cognitive and behavioral skills.

## Limitations

- There could be some variability in the way debriefing is performed, there were two instructors for the execution of the debriefing after each of the scenarios. However, both facilitators were instructors for the neonatal resuscitation program with expertise in debriefing.
- We do not have a control group that has not received debriefing. This is because it has already been widely corroborated in other studies the clear improvement in performance obtained by the participants when receiving the debriefing.

## Additional files

**Additional file 1: Figure S1.** Flowchart of the study design. Source: Prepared by the authors.

**Additional file 2: Appendix S1.** Checklist: neonatal resuscitation performance evaluation tool.
