# Supplementary material for: Evaluation of two strategies for debriefing simulation in the development of skills for neonatal resuscitation: a randomized clinical trial
Source: BMC Res Notes. 2018 Oct 17;11:739. doi: 10.1186/s13104-018-3831-6 (PMC6192222; doi:10.1186/s13104-018-3831-6)
Supplement: Supplementary file 2 — Additional file 2: Appendix S1. Checklist: neonatal resuscitation performance evaluation tool. [file 13104_2018_3831_MOESM2_ESM.docx]

**APPENDIX S1.**

**CHECKLIST: NEONATAL RESUSCITATION PERFORMANCE EVALUATION TOOL**

**NEONATAL RESUSCITATION PERFORMANCE EVALUATION**

Name: __________________________________________ Date: _________

Score: ________________________________________________________

The following is the rubric for the scoring of the specific competency in the development of the simulated scenario. Keep in mind that it consists of eight modules in which objectives and defined cognitive, technical, and behavioral skills are evaluated. The participant will be asked to complete and say each procedure completely, without omitting anything, however insignificant it may seem. Possible scores are: 1 point if you complete the item completely and properly, 0 if you do not do it or if you do it incorrectly or incompletely, and N/A if the item does not apply for the time or its function in the scenario.

Correct - Yes: 1 point (if you complete the item completely and properly)

Incorrect - No: 0 points (if you do not do it or if you do it incorrectly or incompletely)

N/A: does not apply: if the item does not apply for the moment or its function in the scenario.

| **1. Preparation and initial steps** | **YES** | **NO** | **N/A** | **Score** |
| --- | --- | --- | --- | --- |
| Dry and completely remove wet compresses from the newborn |  |  |  | Total score:  ____________  Possible Points:  ____________  Percentage |
| Position the newborn with neck slightly extended |  |  |  |  |
| Remove secretions from the mouth and then the nose with pear syringe |  |  |  |  |
| Gentle and appropriate tactile stimulation |  |  |  |  |
|  | | | | |
| **2. Communicates the heart rate to the resuscitator** | **YES** | **NO** | **N/A** | Total score:  ________  Possible points:  _______  Percentage  _______ |
| Check the heart rate by means of an approved method (stethoscope, palpating cord, or brachial artery) |  |  |  |  |
| Communicates the heart rate to the resuscitator  (beats with the finger, communicates verbally) |  |  |  |  |
|  | | | | |
| **3. Positive pressure ventilation (PPV)** | **YES** | **NO** | **N/A** | **Score** |
| Make the appropriate decision based on the clinical condition of the newborn (apnea, panting, HR < 100) |  |  |  | Total score:  ________  Possible points:  _______  Percentage  _______ |
| Technique: correct HR (40–60 per minute) |  |  |  |  |
| Perform correct pressure and seal (the chest rises properly) |  |  |  |  |
| Properly perform the corrective steps of the PPV |  |  |  |  |
| Re-evaluate by response (HR and color after 30 seconds) |  |  |  |  |
|  | | | | |
| **4. Chest compressions** | **YES** | **NO** | **N/A** | **Score** |
| Make the appropriate decision based on the clinical condition of the newborn (HR < 60 after 30 seconds of effective ventilation) |  |  |  | Total score:  ________  Possible points:  _______  Percentage  _______ |
| Technique: Correct method (two fingers or hands that surround the chest) |  |  |  |  |
| Correct frequency (90 per minute) |  |  |  |  |
| Correct coordination with ventilation (3:1) |  |  |  |  |
| Re-evaluate by response (HR and color after 30 seconds) |  |  |  |  |
|  | | | | |
| **5. Orotracheal intubation** | **YES** | **NO** | **N/A** | **Score** |
| Make the appropriate decision based on the clinical condition of the newborn (prolonged PPV, ineffective bag-mask ventilation, need for medication) |  |  |  | Total score:  ________  Possible points:  _______  Percentage  _______ |
| Technique: correct handling of the laryngoscope |  |  |  |  |
| Correct blade type and number, introduce tube at the correct distance |  |  |  |  |
| Check the position of the endotracheal tube (auscultate, check chest elevation, position colorimetric CO_2_ detector) |  |  |  |  |
| Successful intubation (≤ 2 attempts = Yes, > 2 attempts = No) |  |  |  |  |
|  | | | | |
| **6. Umbilical vein cannulation** | **YES** | **NO** | **N/A** | **Score** |
| Make the appropriate decision based on the clinical condition of the newborn (need for fluids or IV medications) |  |  |  | Total score:  ________  Possible points:  _______  Percentage  _______ |
| Choose the correct catheter number |  |  |  |  |
| Technique: correct insertion technique |  |  |  |  |
| Verify the position (infuses, has blood return) |  |  |  |  |
| Successful cannulation (≤ 2 attempts = Yes, > 2 attempts = No) |  |  |  |  |
|  | | | | |
| **7. Medicines** | **YES** | **NO** | **N/A** | **Score** |
| Proper use of adrenaline (HR < 60 after 30 seconds of PPV and chest compressions) |  |  |  | Total score:  ________  Possible points:  _______  Percentage  _______ |
| Proper dose and administration route |  |  |  |  |
| Correct adrenaline technique: use of 3-way tap, 5-cc bolus given for flushing |  |  |  |  |
| Re-evaluate response |  |  |  |  |
| Proper use of crystalloid. Saline solution or ringer's lactate. (HR < 60 after 30 seconds of PPV and chest compressions, use of adrenaline or history of severe bleeding) |  |  |  |  |
| Proper dose of crystalloids |  |  |  |  |
|  | | | | |
| **8. Behavioral Skills** | **YES** | **NO** | **N/A** | **Score** |
| Check the clinical history |  |  |  | Total score:  ________  Possible points:  _______  Percentage  _______ |
| Based on clinical history, the possible situations were anticipated and planned |  |  |  |  |
| Led the resuscitation team/organize the team/delegate |  |  |  |  |
| Reviewed and prepared the neonatal resuscitation team |  |  |  |  |
| Communicated orders effectively in the team |  |  |  |  |
| Provided feedback to the leader of the completed steps  (HR, Auscultation, medicines, umbilical cannulation) |  |  |  |  |
| Work was delegated optimally |  |  |  |  |
| Used all available resources indicated for the situation |  |  |  |  |
| Requested help when necessary |  |  |  |  |
|  | | | | |
| Total score:  _____________  Possible Score:  _____________  **Percentage (%):**  **______________** | | | | |

PPV: Positive pressure ventilation. HR: Heart rate. IV: Intravenous. Source: Modified from Sawyer T., *et al.* Deliberate Practice Using Simulation Improves Neonatal Resuscitation Performance (18).
